# PD-L1 Inhibitor Cosibelimab for Cutaneous Squamous Cell Carcinoma: Comprehensive Evaluation of Efficacy, Mechanism, and Clinical Trial Insights

**DOI:** 10.3390/biomedicines13040889

**Published:** 2025-04-07

**Authors:** Omer A. Idris, Diana Westgate, Bahar Saadaie Jahromi, Abdulaziz Shebrain, Tiantian Zhang, Hossam M. Ashour

**Affiliations:** 1 Department of Biological Sciences, Western Michigan University, Kalamazoo, MI 49008, USA; omer.a.idris@wmich.edu (O.A.I.); ss.abdulaziz1@gmail.com (A.S.); 2 Malate Institute for Medical Research, Malate Inc., Grandville, MI 49468, USA; 3 Homer Stryker MD School of Medicine, Western Michigan University, Kalamazoo, MI 49008, USA; 4 Forefront Dermatology, Kalamazoo, MI 49007, USA; 5 Department of Hematology and Hematopoietic Stem Cell Transplantation, Toni Stephenson Lymphoma Center, Beckman Research Institute, City of Hope, Duarte, CA 91010, USA; 6 Department of Integrative Biology, College of Arts and Sciences, University of South Florida, St. Petersburg, FL 33701, USA

**Keywords:** cutaneous squamous cell carcinoma, cosibelimab, PD-L1 inhibitors, immunotherapy, neoplasms, biomarkers, combination therapy, immune checkpoint inhibitors, antibody-dependent cellular cytotoxicity (ADCC), clinical trials

## Abstract

Cutaneous squamous cell carcinoma (CSCC) is one of the most common non-melanoma skin cancers, and particularly challenging to treat in advanced or metastatic stages. Traditional therapies, including chemotherapy and radiation, often result in limited efficacy and severe side effects. Cosibelimab, a fully human monoclonal antibody targeting PD-L1, has emerged as a promising immunotherapy for advanced CSCC. In this review, we evaluate the therapeutic potential of cosibelimab by analyzing its mechanism of action, clinical trial data, and its role compared to other PD-1/PD-L1 inhibitors, such as pembrolizumab and cemiplimab. We synthesized the available preclinical and clinical data on cosibelimab, focusing on published Phase I and II trial results involving 76 patients. Objective response rates (ORRs), progression-free survival (PFS), overall survival (OS), and safety profiles were compared between cosibelimab, pembrolizumab, and cemiplimab. Mechanistic insights into cosibelimab’s dual action, including PD-L1 blockade and antibody-dependent cellular cytotoxicity (ADCC), were also explored. Phase II trials demonstrated an ORR of 47.5%, with a median PFS of 12.9 months in advanced CSCC patients. Cosibelimab demonstrated a favorable safety profile, with predominantly mild to moderate adverse events. Comparative analysis with pembrolizumab and cemiplimab showed similar efficacy, although long-term survival data for cosibelimab is still emerging. Given its efficacy and safety, cosibelimab holds promise not only as a monotherapy but also for future exploration in combination regimens and broader oncologic indications. Future trials are required to validate its long-term outcomes, including overall survival, and to explore its use in combination therapies and neoadjuvant/adjuvant settings.

## 1. Introduction

Cutaneous squamous cell carcinoma (CSCC) is the second most common skin cancer worldwide, accounting for approximately 25% of all skin cancers [1]. The incidence of CSCC varies by region, with some areas experiencing high annual rates, as is the case in Northern Australia, where the annual incidence in male patients exceeds 1300 per 100,000 individuals [2]. In the United States, an estimated 1.8 million cases of CSCC are diagnosed annually, contributing to substantial morbidity and healthcare burden [3]. The aggressive nature of CSCC is highlighted by its high morbidity and mortality rates, with advanced-stage CSCC associated with an estimated disease-specific mortality rate of approximately 70% within two years for untreated cases. This underscores the therapeutic challenge, especially in cases of locally unresectable and metastatic CSCC [3]. Several factors contribute to the development of CSCC, including excessive ultraviolet (UV) light exposure, smoking, chronic inflammation, and infection with human papillomavirus (HPV) [2,3].

Current treatment modalities, including surgery, adjuvant radiotherapy, and systemic therapies, have limitations in cases of locally unresectable or metastatic CSCC. Platinum-based chemotherapy, though historically used, is associated with low response rates, high toxicity, and frequent resistance [4]. As a result, immune checkpoint inhibitors (ICIs) targeting the PD-1/PD-L1 pathway, such as pembrolizumab and cemiplimab, have become the preferred systemic treatment options. However, both agents face challenges, including resistance mechanisms and variable responses in immunosuppressed patients.

These challenges underscore the necessity of exploring novel therapeutic options such as ICIs to improve patient prognosis [4]. Currently, the mainstay of treatment for advanced CSCC includes ICIs targeting the PD-1/PD-L1 pathway. Pembrolizumab and cemiplimab are two such agents that have shown efficacy in clinical trials and have received approval for use in cases where surgery or radiation is not feasible. Pembrolizumab, a PD-1 inhibitor, has demonstrated robust anti-tumor activity and is commonly used as a first-line therapy in advanced CSCC, while cemiplimab, another PD-1 inhibitor, has also shown promising results, especially in patients who have not responded to previous therapies. However, both drugs, despite their benefits, have limitations, particularly in addressing tumor heterogeneity and immune evasion mechanisms. As such, there is a growing need for additional agents like cosibelimab, which may offer enhanced efficacy by combining PD-L1 blockade with antibody-dependent cellular cytotoxicity (ADCC), a mechanism that could potentially overcome resistance observed with other checkpoint inhibitors [4]. Unlike PD-1 inhibitors, which prevent T cell exhaustion by blocking interactions with PD-L1 and PD-L2, PD-L1 inhibitors specifically block PD-L1 interactions while preserving PD-L2 signaling, which may reduce immune-related adverse events (irAEs). Additionally, ADCC enhances direct tumor cell killing, potentially improving efficacy in resistant tumors. Comparative evaluation of cosibelimab with existing inhibitors is crucial to determine its efficacy, safety profile, and potential to overcome resistance challenges in CSCC management [4]. Furthermore, individuals who undergo solid organ transplantation are at an increased risk for developing CSCC, largely due to the immunosuppressive agents required to prevent organ rejection. These agents can impair immune surveillance, allowing skin cancer cells to proliferate unchecked [4]. This highlights the need for effective prevention and treatment strategies in this patient population. For example, studies show a decreased risk of new CSCCs with early conversion to sirolimus as a calcineurin inhibitor substitute in transplant recipients [4]. The staging, diagnosis, and management of CSCC have also been a subject of research. The 7th edition of the American Joint Committee on Cancer’s Cancer Staging Manual updated the staging guidelines for CSCC by incorporating evidence-based medicine into the staging process [5]. This evolution of recommendations highlights a need for guidance in identifying the subset of CSCCs that behave aggressively [6]. Immunohistochemical markers, such as IGF-1R, have shown significant association with high-grade CSCC, indicating their potential role in the pathogenesis and progression of the disease [7]. Genetic mutations, particularly in the TP53 gene, play a critical role in the development of CSCC. TP53 mutations are among the most common alterations found in CSCC, contributing to genomic instability and resistance to apoptosis, which are key factors in tumor progression [7]. Emerging evidence suggests that TP53 alterations may also influence the response to ICIs, highlighting the importance of molecular profiling in guiding personalized treatment approaches for CSCC patients. Studies indicate that tumors with high mutational burdens, including those with TP53 mutations, may respond more favorably to ICIs due to the increased likelihood of neoantigen formation [7]. The management of CSCC encompasses a range of treatment modalities, including surgery, adjuvant radiotherapy, and systemic therapies. European consensus-based interdisciplinary guidelines emphasize a multidisciplinary approach to diagnosing and treating invasive CSCC, ensuring optimal patient outcomes [8]. Additionally, studies on treatment patterns and systemic therapies for unresectable locally advanced CSCC (laCSCC) and metastatic CSCC (mCSCC) provide valuable insights into clinical decision-making and improving patient management [9]. Evidence-based analysis of cisplatin, a well-established chemotherapeutic, in CSCC has been previously performed, highlighting the complexities of drug resistance and the need for effective systemic treatment [10]. Additionally, two anti-programmed cell death 1 (anti-PD-1) antibodies, pembrolizumab and cemiplimab, have been approved for managing advanced CSCC when neither radiation nor surgery is a viable treatment option [11,12]. In this review, the basic biology of CSCC is discussed, as well as its treatment options using cosibelimab. Additionally, multiple clinical trials are comprehensively reviewed to evaluate cosibelimab’s therapeutic potential, focusing on trial design, efficacy, safety profile, and duration of response (DOR) in patients with advanced CSCC.

## 2. Basic Biology of CSCC

### 2.1. Cell of Origin

Keratinocyte carcinomas, including CSCC and basal cell carcinoma (BCC), are the most frequently diagnosed cancers in fair-skinned populations [13]. The pathogenesis of CSCC involves a spectrum of malignancies, ranging from actinic keratosis (AK) to metastatic SCC [14]. This progression is driven by cumulative UV-induced DNA damage, which leads to mutations in key tumor suppressor genes such as TP53 and activation of oncogenic pathways. Studies have shown that up to 60% of invasive CSCC cases arise from pre-existing AK lesions, highlighting the significance of early detection and intervention [7,14]. CSCC is primarily derived from keratinocytes, the predominant cell type in the epidermis responsible for producing keratin [15]. Keratinocytes are particularly prone to malignant transformation due to their direct exposure to UV radiation, which induces DNA mutations, impairs DNA repair mechanisms, and promotes chronic inflammation. Additionally, keratinocytes have high proliferative potential and interact with immune cells, making them susceptible to immune evasion and oncogenic transformation [7,14]. Additionally, BCC, squamous cell carcinoma (SCC), and AK all originate from skin keratinocytes [16]. These keratinocyte-derived carcinomas are the most common malignancies in humans [17]. Understanding keratinocyte involvement is essential for advancing CSCC research and treatment.

### 2.2. Genetic Mutations

Genetic mutations play a significant role in the development and progression of CSCC. The TP53 gene, encoding the p53 protein crucial for DNA repair and apoptosis, is frequently mutated in CSCC, leading to uncontrolled cell division and survival [18,19]. Specifically, TP53 mutations are observed in up to 80–90% of CSCC cases, making them the most prevalent genetic alteration compared to other mutations. In contrast, HRAS mutations occur in approximately 10–15% of cases, while NOTCH1 and NOTCH2 mutations are present in 40–60% of cases [20,21,22,23]. Additionally, mutations in the HRAS oncogene can result in continuous cell growth and division, contributing to tumorigenesis [20]. Other key genetic alterations include CDKN2A (p16), NOTCH1, and NOTCH2 mutations, which disrupt cell cycle regulation and differentiation [21,22,23]. Studies have shown that TP53 mutations are highly prevalent in certain subtypes of CSCC, particularly in metastatic or aggressive cases. Exome sequencing has revealed significant levels of TP53 mutations in these instances [24,25]. Furthermore, loss-of-function CDKN2A mutations are also frequently observed in CSCC [21]. The mutation rate of CSCC is notably high, with UV-induced mutations, particularly the C > T or CC > TT UV-signature mutations, being common [19,24]. Moreover, a complex genetic background involving multiple genes and pathways has been implicated in the pathogenesis of CSCC [25]. Additionally, the involvement of genes like TGFBR1, TGFBR2, PIK3CA, and HRAS in CSCC development has also been noted [26,27]. Emerging therapeutic strategies targeting NOTCH mutations include gamma-secretase inhibitors, which block NOTCH receptor activation, and gene-editing techniques such as CRISPR/Cas9 aimed at correcting NOTCH loss-of-function mutations. These approaches are currently under investigation in preclinical models and early-phase clinical trials, showing promise in controlling tumor growth and progression [26,27].

### 2.3. Tumor Development

AK is a precursor lesion to CSCC, presenting as rough, scaly patches on sun-exposed areas [28]. While not all AK progresses to CSCC, the risk of progression increases with the number and size of lesions [29]. AK is indeed considered a neoplastic lesion and precursor to invasive SCC, with a high prevalence and the potential to advance into CSCC [28,30]. Research has shown that CSCC often arises from AK, progressing to invasive primary CSCC, which can metastasize [31]. The genetic landscape of CSCC development involves various biomarkers and gene alterations, with *TP53* mutations as early events in skin carcinogenesis, occurring in approximately 50% of AK cases [32]. Additionally, the upregulation of complement factor D has been identified as a potential biomarker and therapeutic target in CSCC [33]. Identifying significant genes associated with CSCC initiation and development is crucial for understanding the molecular mechanisms underlying tumor progression [34]. Reactive oxygen species (ROS) have been implicated in the proapoptotic effects of certain compounds in CSCC cells, underscoring the importance of oxidative stress in CSCC pathogenesis [30]. Furthermore, MCM2 protein expression has been explored as a proliferative marker in both AK and CSCC, suggesting its potential utility in assessing these skin lesions [35].

### 2.4. Molecular Pathways of CSCC

Multiple signaling pathways play a crucial role in the development and progression of CSCC. The mitogen-activated protein kinase (MAPK)–extracellular signal-regulated kinase (ERK) pathway, involved in cell proliferation and survival, can lead to uncontrolled cell growth when mutated or overactivated [19]. Dysregulation of this pathway often leads to enhanced cell proliferation and resistance to apoptosis, which contributes to the malignant transformation of normal skin cells. The phosphoinositide-3-kinase (PI3K)–protein kinase B/Ak strain transforming (PKB/AKT) pathway, which promotes cell growth, survival, and metabolism, is often dysregulated in various cancers, including CSCC [36,37]. This pathway has been shown to collaborate with the MAPK pathway, amplifying oncogenic signaling and supporting tumor cell survival under adverse conditions. Additionally, NOTCH signaling, typically tumor-suppressive in the skin, can contribute to CSCC when mutated. In normal skin homeostasis, NOTCH signaling controls cell differentiation and proliferation. However, loss-of-function mutations in NOTCH receptors have been identified in both cutaneous and lung SCCs, highlighting the disruption of normal differentiation as a significant mechanism in CSCC pathogenesis [38,39]. These mutations lead to altered differentiation, promoting proliferation and potentially driving malignant transformation. Importantly, research has shown that the crosstalk between the NOTCH and p53 pathways can influence differentiation, apoptosis, and tumorigenesis in CSCC, further complicating the tumor’s molecular landscape [40]. Furthermore, the WNT signaling pathway has emerged as an important player in CSCC development and progression. Aberrant activation of WNT signaling has been implicated in various cancers, including CSCC, where it promotes cell proliferation, migration, and invasion [41]. This pathway’s involvement in regulating cellular responses to oncogenic stress suggests its potential as a therapeutic target in CSCC. Together, these molecular pathways contribute to the aggressive behavior of CSCC, and their complex interactions can influence tumor initiation, progression, and resistance to therapy. Investigating how these pathways crosstalk and how their dysregulation drives tumorigenesis is essential for identifying potential therapeutic targets and strategies to improve outcomes for CSCC patients [41].

### 2.5. Immune Evasion

**Figure 1:**
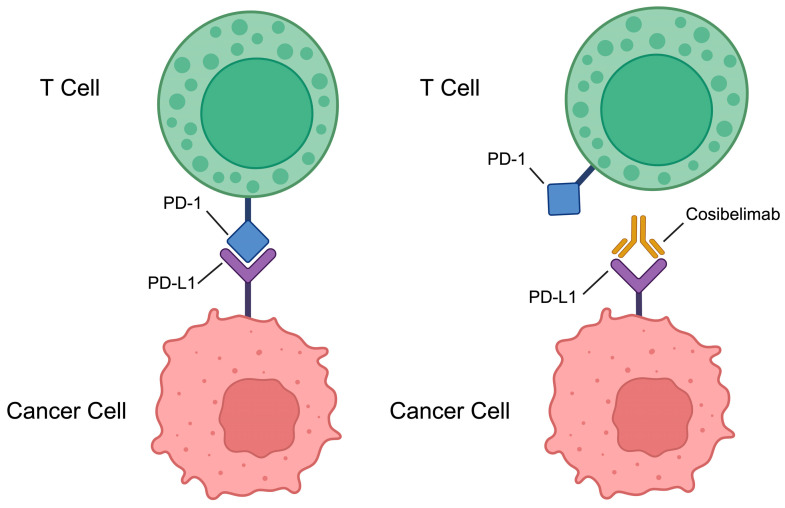
Cosibelimab’s mechanism of action in blocking PD-1/PD-L1 interactions in CSCC (Created with Biorender.com). This figure illustrates the immune checkpoint interaction between PD-1 on T cells and PD-L1 on tumor cells. Cosibelimab, a PD-1 inhibitor, binds to PD-1 on T cells, blocking its interaction with PD-L1 on tumor cells. This inhibition prevents tumor immune evasion and promotes an anti-tumor immune response.

CSCC utilizes various mechanisms to evade the immune system, promoting tumor growth and progression. One key strategy involves the expression of programmed death-ligand 1 (PD-L1) on tumor cells, which interacts with PD-1 receptors on T cells, leading to T cell inhibition and immune evasion [42]. The tumor microenvironment in CSCC often contains immunosuppressive cells, such as regulatory T cells (Tregs) and myeloid-derived suppressor cells (MDSCs), which further inhibit anti-tumor immune responses [43]. These evasion tactics contribute to the aggressive nature of CSCC and are associated with a poor prognosis [44]. Studies have shown that PD-L1 expression is increased in CSCC, indicating a potential immune evasion mechanism [45]. However, the response to immune checkpoint blockade (ICB) therapies targeting PD-L1 in CSCC is limited, with only a fraction of patients responding and many experiencing tumor recurrences due to immune evasion mechanisms [46]. The expression of PD-L1 and other immune-inhibitory molecules in the tumor microenvironment highlights the role of immune evasion in CSCC [47]. Furthermore, PD-L1 upregulation has been associated with a high response rate to PD-1 inhibitors in patients with advanced CSCC, highlighting the potential of targeting immune checkpoints [48]. PD-L1 expression is linked to adverse outcomes in various malignancies, emphasizing its role in promoting tumor progression and immune evasion [49]. Targeting these immune evasion pathways with specific therapies, such as cosibelimab, represents a promising approach in managing advanced CSCC. Cosibelimab’s role in blocking the PD-1/PD-L1 interaction and preventing immune evasion in CSCC is illustrated in Figure 1.

### 2.6. Standard Treatment for CSCC

Treatment strategies for CSCC encompass a range of modalities tailored to the disease stage and patient characteristics. The primary treatment for localized CSCC involves surgical excision with clear margins to ensure complete removal, which typically results in good prognosis and cure rates exceeding 90% [23,50,51]. In cases where surgical excision is not feasible or for high-risk lesions, radiation therapy can be employed either as a primary treatment for inoperable tumors or as adjuvant therapy to reduce the risk of recurrence [52,53]. Surgical excision remains the standard of care for high-risk CSCC, with the additional resection of positive margins when possible [54]. For advanced cases of CSCC, systemic therapies play a crucial role in managing the disease. Chemotherapy, such as cisplatin, targeted therapies like EGFR inhibitors (e.g., cetuximab), and immunotherapy with PD-1 inhibitors (e.g., pembrolizumab) are used against advanced or metastatic CSCC [55,56,57]. Immunotherapy, particularly with PD-1 inhibitors, has shown promising results in treating locally advanced, metastatic, or relapsed CSCC, with significant objective response rates (ORRs) and disease control rates [57,58].

While surgery remains the cornerstone of CSCC treatment, especially in the early stages, the emergence of immunotherapies has provided new avenues for managing advanced disease. Understanding the role of each treatment modality and tailoring therapy based on disease characteristics and patient factors is essential in optimizing outcomes for individuals with CSCC. As the management of advanced CSCC evolves, the approval of novel immunotherapy agents like cosibelimab represents a significant advancement in treatment options.

## 3. Cosibelimab for CSCC

Cosibelimab, a fully human monoclonal antibody that targets PD-L1, has emerged as a promising therapeutic option for CSCC, particularly in advanced or metastatic cases. CSCC is one of the most prevalent forms of non-melanoma skin cancer, with early-stage disease often manageable through surgical excision. However, advanced or metastatic CSCC presents significant treatment challenges, as traditional therapies such as chemotherapy and radiation have limited efficacy and are frequently accompanied by severe side effects [59]. The advent of immunotherapy, particularly agents that inhibit the PD-1/PD-L1 pathway, has revolutionized treatment options for these patients, offering a more targeted approach that can enhance the immune system’s ability to combat cancer [60,61]. Recent clinical studies have demonstrated that cosibelimab can yield favorable outcomes in patients with locally advanced and metastatic CSCC, marking it as a viable alternative to conventional therapies [62]. The clinical success of cosibelimab not only highlights the growing role of ICIs in the management of skin cancers but also underscores the need for continued research into novel immunotherapeutic strategies [63]. The efficacy of cosibelimab in enhancing anti-tumor responses illustrates the potential of PD-1/PD-L1 inhibitors to improve patient outcomes in the context of aggressive skin malignancies [64].

### 3.1. Cosibelimab Mechanism of Action

Cosibelimab operates through a dual mechanism of action, making it a unique and potentially more effective ICI. At its core, cosibelimab blocks the interaction between PD-L1 expressed on tumor cells and the PD-1 receptor on T cells. This PD-1/PD-L1 interaction is a well-known immune evasion strategy employed by many cancers, including CSCC. Under normal conditions, the binding of PD-L1 to PD-1 dampens T cell activation, allowing tumor cells to escape immune surveillance and continue proliferating. By inhibiting this interaction, cosibelimab reactivates the immune system, enabling effective tumor cell elimination [65]. In addition to blocking the PD-1/PD-L1 pathway, cosibelimab has the potential to trigger ADCC, a mechanism that is often overlooked in discussions of ICIs. ADCC involves the activation of natural killer (NK) cells, key players in the innate immune response. When cosibelimab binds to PD-L1 on tumor cells, it can also engage NK cells through the Fc region of the antibody. This interaction leads to the direct lysis of tumor cells by NK cells, providing an additional layer of immune activation. This mechanism is particularly significant because NK cells can recognize and eliminate tumor cells independently of T cell infiltration, which is crucial in tumors with low T cell activity or those that exhibit resistance to T cell-mediated immune responses [65]. The therapeutic significance of ADCC lies in its ability to overcome resistance mechanisms that may limit the effectiveness of traditional ICIs. In tumors where T cell responses are suboptimal due to low T cell infiltration or immune escape mechanisms, ADCC offers an alternative pathway for targeting and killing tumor cells. Additionally, ADCC may be beneficial in cases where PD-L1 expression is high on tumor cells, but T cell responses are weak. This dual mechanism, combining PD-1/PD-L1 blockade and ADCC, may offer superior outcomes compared to traditional PD-1 inhibitors by providing a broader and more sustained anti-tumor response, especially in tumors with poor T cell infiltration or immune evasion. Early studies suggest that cosibelimab’s dual functionality—engaging both the adaptive immune system via PD-1/PD-L1 blockade and the innate immune system via ADCC—could result in more robust and sustained anti-tumor responses, particularly in challenging cases where other therapies might fail [65].

**Figure 2:**
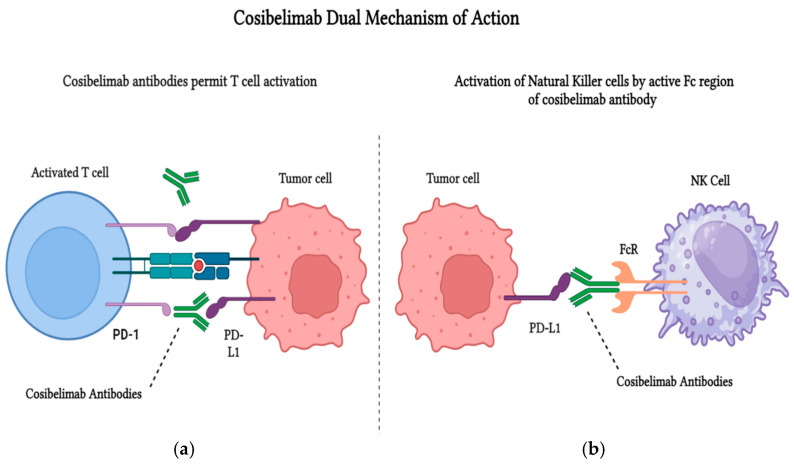
The dual mechanism of action of cosibelimab (Created with Biorender.com). (**a**) Cosibelimab stimulates T cell activation by blocking the interaction between PD-1 on T cells and PD-L1 on tumor cells, preventing tumor immune evasion. (**b**) Activation of natural killer (NK) cells by the active Fc region of cosibelimab may contribute to NK-mediated tumor cell killing, although this remains under investigation for its role in solid tumors.

However, patient response to ADCC can vary depending on factors such as the expression levels of Fc receptors on immune effector cells, the degree of tumor heterogeneity, and the immunosuppressive nature of the tumor microenvironment [65]. Beyond its role in tumor cell destruction, the therapeutic significance of ADCC lies in its potential to overcome resistance mechanisms observed with traditional ICIs. However, patient response to ADCC can vary significantly based on factors such as the expression levels of Fc receptors on immune effector cells, tumor heterogeneity, and the immunosuppressive nature of the tumor microenvironment [65]. Understanding these variables is crucial for optimizing patient selection and developing personalized treatment strategies that harness the full potential of cosibelimab’s dual mechanism [65]. The variation in patient response highlights the importance of understanding these factors for optimizing patient selection and maximizing the efficacy of cosibelimab. As ongoing clinical trials continue to evaluate cosibelimab, its unique mechanism, as depicted in Figure 2, remains a critical area of research, offering new insights into how ICIs can be optimized to maximize patient outcomes [65].

### 3.2. Approval Status of Cosibelimab for CSCC

Cosibelimab, a fully human monoclonal antibody targeting PD-L1, has shown promising results in clinical trials for treating CSCC, particularly in advanced or metastatic cases. While it was previously undergoing clinical evaluation, cosibelimab received FDA approval on 13 December 2024, for adults with laCSCC or mCSCC who are not candidates for radiation therapy or curative surgery [65]. This approval marks a significant step forward in the treatment of advanced CSCC, as it provides a new option for patients with limited alternatives [3,65]. Cosibelimab’s approval process followed a detailed timeline. The FDA initially received a Biologics License Application (BLA) for cosibelimab in January 2023. However, in December 2023, the FDA issued a complete response letter due to issues with a third-party manufacturing facility. The BLA was resubmitted in July 2024, and the FDA set a Prescription Drug User Fee Act date of 28 December 2024 to make a decision. Ultimately, cosibelimab received FDA approval based on results from a Phase I clinical trial, which enrolled patients with metastatic or laCSCC who were not candidates for surgery or radiation. These trials demonstrated favorable efficacy and safety profiles, underscoring cosibelimab’s potential to significantly improve patient outcomes in this population [66,67]. Prior to this approval, cosibelimab had been granted Fast Track designation by the FDA, aimed at expediting the development and review of drugs addressing unmet medical needs in serious conditions. The Fast Track process typically involves rolling reviews, frequent interactions with the FDA, and accelerated approval procedures, all designed to expedite access to treatments for serious conditions. This designation highlighted cosibelimab’s potential to offer substantial therapeutic benefits for patients suffering from advanced CSCC, a population often facing aggressive disease and limited treatment options [65]. Additionally, cosibelimab has been evaluated under the FDA’s Orphan Drug designation, further emphasizing its potential in treating rare or difficult-to-manage cancers [65]. Cosibelimab’s regulatory status contrasts with other PD-L1 inhibitors such as pembrolizumab and cemiplimab. Pembrolizumab, approved by the FDA for advanced CSCC in 2018, has a well-established track record but primarily targets PD-1 rather than PD-L1. Cemiplimab, also a PD-1 inhibitor, was approved by the FDA in 2020 for patients with advanced CSCC, offering another treatment option for similar patient populations. Cosibelimab’s recent approval adds to these options, expanding the treatment arsenal for advanced CSCC patients with unmet needs.

### 3.3. Inclusion and Exclusion Criteria for Cosibelimab Trials: Phase I and Phase II

The inclusion and exclusion criteria for the Phase I and Phase II clinical trials of cosibelimab provide essential insights into patient eligibility and the populations studied (Table 1). These criteria ensure the safety and appropriateness of participants for the respective trial phases. Additionally, subgroup analyses conducted during these trials offer valuable information on the differential responses among various patient groups. The following table summarizes the key inclusion and exclusion criteria, as well as the subgroup analysis for both trial phases.

### 3.4. Evidence on Cosibelimab from Phase II Clinical Trial

Cosibelimab has shown significant promise in the Phase II clinical trial specifically designed for patients with advanced CSCC. This trial primarily aimed to assess the safety, efficacy, and tolerability of cosibelimab as a monotherapy for individuals with locally advanced or metastatic CSCC who had limited treatment options. By targeting PD-L1, cosibelimab leverages both immune checkpoint blockade and potential activation of NK cells, positioning it as a noteworthy candidate in the immunotherapy landscape [65,68].

#### 3.4.1. Cosibeliamb Phase II Clinical Trial Study Design

The Phase II clinical trial of cosibelimab was structured as a multicenter, open-label study focusing on patients with locally advanced or metastatic CSCC who were not candidates for curative surgery or radiation therapy. Participants received a fixed dose of cosibelimab every two weeks, with tumor responses evaluated using the Response Evaluation Criteria in Solid Tumors (RECIST) guidelines through imaging techniques such as CT or MRI [65,67]. The primary endpoint was the ORR, which measures the proportion of patients whose tumors shrink or disappear following treatment. Secondary endpoints included progression-free survival (PFS), overall survival (OS), DOR, and safety, with a particular emphasis on immune-mediated adverse events [68]. An exploratory analysis was also conducted to assess the correlation between PD-L1 expression and treatment efficacy, although cosibelimab demonstrated activity regardless of PD-L1 status, indicating its broad applicability across diverse patient subgroups [65,68].

#### 3.4.2. Efficacy of Cosibelimab

The results from the Phase II clinical trial highlighted the strong efficacy of cosibelimab in treating advanced CSCC. The trial reported an ORR of 47.5%, with 7% of patients achieving complete responses (CR) and 40.5% achieving partial responses (PR) [66,67]. This indicates that nearly half of the patients experienced significant tumor reduction or elimination. The median PFS was reported at 12.9 months, suggesting that patients maintained control over their disease for over a year on average. Furthermore, the median DOR was 11.3 months, underscoring the long-lasting nature of the therapeutic effects once a response was achieved. Early OS data indicated a median OS of 18.4 months, suggesting an improvement in life expectancy for this challenging patient population [66,67]. Notably, the efficacy of cosibelimab was observed regardless of PD-L1 expression levels, reinforcing its potential as a therapeutic option across various patient demographics [65,68].

#### 3.4.3. Cosibelimab’s Safety

In terms of safety, the Phase II clinical trial demonstrated a favorable profile for cosibelimab in patients with advanced CSCC. The majority of adverse events were mild to moderate in severity (Grade 1 or 2), with the most common treatment-related events being fatigue, rash, and pruritus [65,68]. Severe adverse events (Grade 3 or higher) were relatively infrequent and included immune-related conditions such as pneumonitis and dermatitis, which were effectively managed with medical interventions like corticosteroids. Importantly, no new safety concerns emerged during the trial, and the incidence of irAEs was consistent with expectations for PD-L1 inhibitors [65,68]. Treatment discontinuation due to adverse events was rare, highlighting the overall tolerability of cosibelimab. Furthermore, no treatment-related deaths were reported, and serious adverse events were uncommon, suggesting that cosibelimab is both effective and well-tolerated, supporting its potential as a viable treatment option for patients with advanced CSCC [65,68].

## 4. Discussion

### 4.1. Cosibelimab in Other Advanced Cancers: Evidence from Phase I and Broader Phase II Trials

Cosibelimab has been evaluated in a variety of advanced cancers, including CSCC and non-small cell lung cancer (NSCLC), across both the Phase I and broader Phase II clinical trials. Each trial provided distinct insights into the drug’s safety, tolerability, pharmacokinetics, and efficacy, shaping the current understanding of cosibelimab’s therapeutic potential across multiple cancer types. Below, the design, efficacy, and safety of these two trials are discussed, highlighting their contributions to the development of cosibelimab [65,68].

#### 4.1.1. Phase I Trial

##### Study Design

The Phase I trial was primarily designed to assess the safety, tolerability, and pharmacokinetics of cosibelimab in patients with advanced solid tumors, including CSCC. This multicenter, dose-escalation study aimed to determine the recommended dose for future studies. Patients with various advanced cancers were enrolled and received cosibelimab at escalating doses. The trial followed a standard 3 + 3 design, focusing on identifying dose-limiting toxicities (DLTs) and gathering preliminary data on anti-tumor activity [65,69,70].

##### Efficacy

While the primary focus of the Phase I trial was safety, preliminary efficacy data were also collected. Cosibelimab demonstrated early signs of anti-tumor activity, with several patients achieving PRs or stable disease, particularly in those with PD-L1-positive tumors. In the metastatic CSCC cohort of 78 participants, the ORR was 47.4% (95% CI: 36.0% to 59.1%), with a median follow-up of 15.4 months (range: 0.4 to 40.5 months). The median DOR was not reached, and 73.0% of patients showed ongoing responses. Although the ORR was modest, these findings provided a foundation for further exploration of cosibelimab’s efficacy in subsequent trials [65,71,72].

##### Safety

The safety data from the Phase I trial indicated that cosibelimab was generally well-tolerated across different dose levels. The most common adverse events were mild to moderate (Grade 1 or 2), including fatigue (26.9%), rash (16.7%), and anemia (15.4%). Serious irAEs, such as pneumonitis or colitis, were observed infrequently, with 23.1% of participants experiencing irAEs (Grade 3: 2.6%), and were effectively managed with corticosteroids. No treatment-related deaths were reported. DLTs were rare, and no maximum tolerated dose (MTD) was reached, allowing for the selection of a dose for the broader Phase II trial. This trial established the safety and tolerability of cosibelimab, providing a basis for its further development [65,69,71].

#### 4.1.2. Broader Phase II Trial

##### Study Design

The broader Phase II trial was designed to assess the efficacy of cosibelimab across a range of advanced solid tumors, including CSCC, NSCLC, and other cancers. This multicenter, open-label study focused on evaluating the drug’s anti-tumor activity, with key endpoints including ORRs, PFS, and OS. Unlike the Phase I trial, which concentrated on safety and dose determination, the broader Phase II trial aimed to establish the clinical effectiveness of cosibelimab across a broader patient population [65,73,74].

##### Efficacy

In the broader Phase II trial, cosibelimab demonstrated significant efficacy in multiple tumor types, particularly in patients with PD-L1-positive cancers. In NSCLC, the ORR reached approximately 40% in patients with high PD-L1 expression, while in CSCC, the ORR mirrored earlier findings from other trials at around 47% [71,72]. The PFS and OS metrics were favorable, particularly for patients with higher PD-L1 expression levels. Responses were durable across various cancer types, making cosibelimab a compelling option for advanced solid tumors beyond skin cancers [4,65,75].

##### Safety

The safety profile in the broader Phase II trial was consistent with earlier findings from the Phase I study. Most adverse events were mild to moderate (Grade 1 or 2) and included fatigue, rash, and pruritus. Severe irAEs (Grade 3 or higher), such as pneumonitis and colitis, were reported but were infrequent and managed effectively with immunosuppressive therapies. No new safety signals emerged during the broader Phase II trial, and treatment discontinuation due to adverse events remained low. These findings confirmed cosibelimab’s favorable safety profile across a range of solid tumors, reinforcing its potential as a broad-spectrum immunotherapy [65,72,76].

### 4.2. Comparative Efficacy and Safety of Cosibelimab Across Various Types of Cancers: Insights from Clinical Trials

The clinical evaluation of cosibelimab in CSCC across multiple trials, including the CSCC-specific Phase II trial, the broader Phase II trial, and the Phase I trial, provides valuable insights into its efficacy and safety profile. By comparing the findings across these studies as shown in Table 2, we can better understand how cosibelimab performs in different settings, its potential benefits, and the consistency of its therapeutic outcomes.

#### 4.2.1. Efficacy Comparison

The similarity in efficacy between the CSCC cohort in the broader Phase II trial and the dedicated CSCC Phase II trial supports the consistency of cosibelimab’s therapeutic benefit in this cancer type. Furthermore, the broader trial demonstrated that cosibelimab demonstrated efficacy irrespective of PD-L1 expression levels, confirming the drug’s versatility [77]. The Phase I trial primarily focused on safety, but it did report preliminary efficacy data, showing modest anti-tumor activity in a small number of patients with CSCC. Although the ORR was not as robust as in the later-phase trials, early signs of PRs and disease stabilization supported the continued development of cosibelimab and paved the way for more definitive Phase II studies [58,65]. Collectively, these findings highlight cosibelimab’s substantial and consistent anti-tumor activity in CSCC, particularly in patients with advanced or metastatic disease. Additionally, cosibelimab’s potential for ADCC could contribute to better long-term outcomes. ADCC may help in sustaining immune responses against tumor cells by facilitating the killing of tumor cells through immune cell activation, even beyond the immediate treatment phase. This mechanism could result in prolonged disease control and possibly improve long-term survival in patients with advanced CSCC. The observed ORR of around 47% across the Phase II trials, along with durable responses, positions cosibelimab as a highly effective treatment option for CSCC, with promising outcomes that are comparable across different study designs [65].

#### 4.2.2. Safety Comparison

The safety profile of cosibelimab has been remarkably consistent across all trials, with most adverse events being mild to moderate (Grade 1 or 2) in severity. In the CSCC-specific Phase II trial, the most common treatment-related adverse events were fatigue, rash, and pruritus, all of which were manageable and generally did not result in treatment discontinuation [65,67]. Severe adverse events (Grade 3 or higher) were rare, occurring in less than 10% of patients. The most significant irAEs were pneumonitis and immune-mediated dermatitis, but these were effectively managed with standard immunosuppressive treatments such as corticosteroids [65,67]. In the broader Phase II trial, similar safety outcomes were reported, with fatigue, pruritus, and rash again being the most frequent mild-to-moderate side effects. irAEs, such as colitis, hepatitis, and pneumonitis, were observed in a small subset of patients but were managed with treatment pauses or corticosteroids. Importantly, no new safety concerns arose in this broader cohort, further confirming cosibelimab’s manageable safety profile even when used across multiple tumor types [77]. The Phase I trial, being an early-stage study, focused heavily on safety and DLTs. The results from this trial confirmed cosibelimab’s favorable safety profile, with no maximum tolerated dose (MTD) reached and a low incidence of severe adverse events. The dose established in this trial was subsequently used in the Phase II studies, which maintained similar safety outcomes [58]. When comparing across the trials, the safety data remain highly consistent, indicating that cosibelimab is a well-tolerated treatment option for patients with advanced CSCC. The incidence of serious adverse events is low, and most side effects can be managed with standard interventions, such as corticosteroids. This consistency in safety, observed from early-phase trials through to more expansive studies, strengthens the confidence in cosibelimab as a viable treatment for patients with advanced CSCC, with a predictable and manageable safety profile [65].

### 4.3. Comparative Analysis of Cosibelimab, Pembrolizumab, and Cemiplimab in CSCC

Immunotherapy for CSCC has advanced with the introduction of cosibelimab, pembrolizumab, and cemiplimab. Although all three agents target the PD-1/PD-L1 pathway, their mechanisms of action, efficacy, safety profiles, and practical aspects offer important differences and similarities, as shown in Table 3, providing clinicians with options for personalized treatment.

#### 4.3.1. Mechanism of Action

##### Similarities

Cosibelimab, pembrolizumab, and cemiplimab all function by targeting the PD-1/PD-L1 immune checkpoint pathway, thereby restoring T cell activity to attack tumor cells. Pembrolizumab and cemiplimab inhibit the PD-1 receptor on T cells, blocking its interaction with PD-L1 and PD-L2 ligands expressed by tumor cells. This blockade reactivates T cells, enabling them to detect and destroy cancer cells that had previously evaded immune surveillance. Cosibelimab, while also inhibiting the PD-1/PD-L1 pathway, binds directly to PD-L1 on tumor cells, preventing it from interacting with PD-1 on immune cells. This mechanism similarly reactivates T cells and restores immune surveillance. The fundamental similarity across all three agents is their goal of neutralizing the immune escape mechanisms of cancer cells through modulation of the PD-1/PD-L1 axis [58,65].

##### Differences

A notable distinction is cosibelimab’s potential to engage ADCC through its Fc region. This allows for the possible activation of NK cells, adding another layer of immune response beyond T cell activation, which is not a feature of pembrolizumab or cemiplimab. The ADCC mechanism offers cosibelimab the potential for enhanced tumor cell killing by engaging both the adaptive and innate immune systems. In contrast, pembrolizumab and cemiplimab rely solely on T cell-mediated immune responses and do not engage NK cells via ADCC [65].

#### 4.3.2. Efficacy Comparison

##### Objective Response Rates

The efficacy of all three agents is comparable in advanced CSCC, with cosibelimab, pembrolizumab, and cemiplimab each demonstrating ORRs in the 40–50% range. In its Phase II trial, cosibelimab achieved an ORR of 47.5%, with 7% of patients achieving CR. Similarly, cemiplimab reported an ORR of 47%, but with a slightly higher CR rate of 13%. Pembrolizumab’s ORR for CSCC also falls within the 40–50% range, though CR rates tend to be slightly lower compared to cemiplimab [58,78].

##### Duration of Response and Progression-Free Survival

When examining the DOR and PFS, cemiplimab shows a slight advantage, with a median DOR of 16.8 months and a median PFS of 18 months. In comparison, cosibelimab’s DOR is 11.3 months, with a PFS of 12.9 months, slightly shorter but still offering durable disease control. Pembrolizumab demonstrates similar outcomes in PFS and DOR, comparable to cemiplimab but generally aligned with patient PD-L1 expression levels [58,78].

##### Efficacy Across PD-L1 Expression Levels

While all three agents show efficacy across PD-L1 expression levels, pembrolizumab tends to perform better in PD-L1-high expressors (≥50%). Both cemiplimab and cosibelimab have demonstrated activity regardless of PD-L1 status, making them viable options even in patients with lower PD-L1 expression [65].

#### 4.3.3. Safety Comparison

##### Common Adverse Events

The safety profiles of cosibelimab, pembrolizumab, and cemiplimab are broadly similar, with the most common adverse events being mild to moderate immune-related side effects such as fatigue, rash, pruritus, and diarrhea. These events typically fall within Grade 1 or 2 in severity [65].

##### Severe Immune-Related Adverse Events

One distinction is that cosibelimab demonstrated a slightly lower incidence of Grade 3 or higher adverse events in its Phase II trial, occurring in fewer than 10% of patients. In contrast, both pembrolizumab and cemiplimab report 10–15% of patients experiencing severe irAEs, such as pneumonitis, colitis, and endocrinopathies like thyroid dysfunction and adrenal insufficiency. Cemiplimab has been associated with a higher incidence of endocrinopathies compared to the other two agents, requiring more careful monitoring of thyroid and adrenal function during treatment. Pembrolizumab shares similar risks of irAEs, particularly in patients with pre-existing autoimmune conditions, which require close monitoring and potentially early intervention with corticosteroids [65].

##### Management of Adverse Events

All three agents necessitate careful management of irAEs, often with corticosteroids or other immunosuppressants. Cosibelimab, with its lower rate of severe events, may offer a slight safety advantage, particularly for patients at higher risk of severe irAEs or those with underlying health conditions [65].

#### 4.3.4. Practical Considerations

##### Dosing Schedules

Dosing convenience is a key consideration. Pembrolizumab offers flexible dosing schedules, including administration every 3 or 6 weeks, which can be particularly beneficial for patients who prefer less frequent clinic visits. Cemiplimab is administered every 3 weeks, while cosibelimab is dosed every 2 weeks. Though cosibelimab requires more frequent administration, this may be advantageous for patients needing closer monitoring or with more aggressive disease [65].

##### Availability and Use in Clinical Practice

Cemiplimab was the first PD-1 inhibitor approved specifically for CSCC and has gained wide acceptance in clinical practice. Pembrolizumab, while approved for multiple cancer types including CSCC, is already widely available and has an established use in immunotherapy. Cosibelimab, though not yet approved for widespread use, shows potential to become a competitive option once fully evaluated and available in the clinical setting [65].

#### 4.3.5. Comparison of Long-Term Survival Data

The clinical landscape for treating advanced CSCC has been significantly shaped by the introduction of PD-1/PD-L1 inhibitors, including pembrolizumab and cemiplimab. Each of these agents demonstrates robust efficacy, but long-term survival data, including OS and PFS, provide a deeper understanding of their potential to deliver sustained benefits. The long-term data for pembrolizumab and cemiplimab indicate that both agents provide extended survival benefits, particularly for patients with advanced disease. While cosibelimab’s long-term outcomes are not yet fully characterized, the early response rates and PFS suggest a favorable trajectory. Future research should focus on verifying whether the potential engagement of NK cells via ADCC in cosibelimab contributes to more sustained responses, possibly offering advantages in the long-term control of advanced CSCC [3].

##### Pembrolizumab

Pembrolizumab, evaluated in the KEYNOTE-629 trial, reported promising long-term outcomes for advanced CSCC patients. The trial indicated a median OS of 22 months and a median PFS extending to 18.4 months, demonstrating pembrolizumab’s capacity to provide durable responses in a substantial proportion of patients. Notably, patients with high PD-L1 expression exhibited enhanced survival outcomes, underscoring the relevance of biomarker-driven therapies in long-term management [58].

##### Cemiplimab

Cemiplimab, evaluated in the EMPOWER-CSCC 1 trial, demonstrated comparable long-term efficacy, with a median OS of 19 months and a median PFS of 16.8 months. Cemiplimab achieved an ORR of 47%, with durable responses observed across both high and low PD-L1 expressors. This establishes cemiplimab as a consistent option for long-term disease control [67].

##### Cosibelimab

Cosibelimab has shown promising early efficacy in clinical trials, but comprehensive long-term survival data are still lacking. Its dual mechanism of PD-L1 blockade and potential ADCC may contribute to durable responses. Ongoing trials will be crucial to confirm whether cosibelimab can achieve long-term outcomes similar to or surpassing those of pembrolizumab and cemiplimab [65].

### 4.4. Future Directions

Cosibelimab has shown significant promise in the treatment of advanced CSCC, but there are several areas of future research and development that could further optimize its clinical utility and expand its therapeutic potential.

#### 4.4.1. Biomarker-Driven Personalization

While PD-L1 expression has been a commonly used biomarker to predict responses to PD-1/PD-L1 inhibitors, including cosibelimab, its predictive power is not absolute. Emerging evidence suggests that a multifaceted approach involving additional biomarkers could improve patient stratification for cosibelimab therapy. Tumor mutational burden (TMB) has garnered attention as a potential biomarker, given its association with the generation of neoantigens that enhance immune recognition. Higher TMB may correlate with improved responses to PD-1/PD-L1 inhibitors, though its role in CSCC specifically warrants further exploration. The density and functionality of tumor-infiltrating lymphocytes (TILs) within the tumor microenvironment also hold promise as predictive markers [79,80]. Elevated TIL densities are often indicative of a pre-existing antitumor immune response, which may be reinvigorated by ICIs like cosibelimab. Variability in human leukocyte antigen (HLA) genes has been linked to differences in antigen presentation and immune system activation. Patients with broader HLA diversity may exhibit better responses to immunotherapy, though more research is needed to validate this finding in CSCC. In addition to TMB, TILs, and HLA, other potential biomarkers to consider for patient stratification include LAG-3, TIM-3, and circulating tumor DNA, which may provide real-time insights into disease progression and treatment response. LAG-3 and TIM-3 are immune checkpoint molecules that could help identify patients likely to benefit from cosibelimab, especially those whose tumors exhibit resistance mechanisms due to alternative immune checkpoint pathways. Systemic biomarkers, such as circulating immune cells or cytokine levels, provide a non-invasive means to monitor treatment responses. Similarly, the gut microbiome’s influence on systemic immunity suggests that its composition could modulate patient outcomes, with evidence pointing to certain microbial profiles enhancing immunotherapy efficacy. Future research should prioritize the integration of these biomarkers into clinical trials for cosibelimab. A comprehensive understanding of these factors could enable clinicians to personalize therapy, improving efficacy and minimizing unnecessary treatment in patients less likely to respond [79,80].

#### 4.4.2. NK Cell Activation and Resistance Mechanisms in Tumor Immunotherapy

NK cells are essential in the body’s immune response to tumors, particularly through their ability to recognize and destroy malignant cells. The activation of NK cells is triggered by several factors, including the presence of altered or stressed cells and the tumor microenvironment, which can either promote or inhibit NK cell function. Tumors, however, often develop strategies to evade NK cell-mediated cytotoxicity, such as the upregulation of immune checkpoint molecules like PD-L1 [79,80]. This interaction with PD-1 on NK cells can inhibit their activity, limiting the effectiveness of ICIs like cosibelimab. Additionally, resistance to immunotherapy can result from the presence of other immunosuppressive mechanisms, including the accumulation of Tregs and MDSCs, which dampen NK cell activation and function. Moreover, tumor heterogeneity and the dynamic evolution of immune escape mechanisms pose significant challenges in achieving long-term therapeutic success. Addressing these resistance mechanisms and understanding how they impact NK cell activation and clinical outcomes could enhance the long-term efficacy of PD-1 inhibitors. Future strategies may focus on improving NK cell responses through combination therapies or by targeting these immune resistance pathways, ultimately contributing to better treatment outcomes [79,80].

#### 4.4.3. Combination Strategies

Resistance to ICIs can arise due to both intrinsic and acquired mechanisms, which are often shared across tumor types and not exclusive to CSCC. Intrinsic resistance may stem from tumor-intrinsic factors such as low TMB, lack of neoantigen presentation, or defective interferon-gamma signaling pathways [81,82]. Acquired resistance, on the other hand, can develop during treatment due to adaptive immune evasion, such as upregulation of alternative immune checkpoints (e.g., LAG-3, TIGIT) or depletion of effector T cells in the tumor microenvironment. These resistance mechanisms highlight the need for innovative therapeutic strategies to enhance the efficacy of ICIs. The potential of combination therapies with cosibelimab warrants exploration in this context. Given its ability to engage both T cell activation and potential ADCC via NK cells, combining cosibelimab with other ICIs, such as CTLA-4 inhibitors or newer agents targeting LAG-3 or TIGIT, could synergistically overcome resistance mechanisms and improve anti-tumor immune responses. Additionally, combining cosibelimab with conventional treatments, such as radiotherapy or targeted therapies, may re-sensitize resistant tumors or enhance its effectiveness in aggressive forms of CSCC [81,82].

#### 4.4.4. Overcoming Resistance

Despite cosibelimab’s demonstrated efficacy in clinical trials, some patients experience primary or acquired resistance to PD-L1 blockade. Understanding the underlying mechanisms of this resistance—whether through changes in the tumor microenvironment, immune system evasion, or molecular alterations—could lead to the development of new strategies to overcome resistance. This may involve combining cosibelimab with agents that modulate the tumor microenvironment or directly target resistance pathways [83,84].

#### 4.4.5. Investigating ADCC Potential

One unique aspect of cosibelimab is its potential to activate ADCC through NK cell engagement, which could provide an additional mechanism of tumor destruction beyond PD-L1 inhibition. Preclinical studies using xenograft and syngeneic mouse models could help further investigate this potential, particularly in PD-L1-low tumors, where ADCC may play a more prominent role. In addition, exploring the combination of cosibelimab with NK cell-activating agents or monoclonal antibodies targeting tumor-specific antigens may help optimize its ADCC activity. Understanding the mechanisms by which cosibelimab activates NK cells and whether this effect varies across tumor types will broaden its therapeutic application [85,86].

#### 4.4.6. Expanding Applications Beyond CSCC

While cosibelimab has shown efficacy in CSCC, ongoing and future clinical trials should explore its use in other solid tumors, especially those with high PD-L1 expression or where NK cell activity might play a role in tumor control. The versatility of cosibelimab’s dual immune engagement suggests that it could be effective beyond skin cancers, potentially expanding its indications in the future [87,88].

#### 4.4.7. Optimizing Dosing Schedules

Given cosibelimab’s every 2-week dosing schedule, future research could focus on optimizing dosing regimens to enhance patient convenience while maintaining efficacy. Investigating longer-acting formulations or personalized dosing strategies based on patient-specific factors, such as tumor burden or immune system status, could reduce the frequency of treatment visits without compromising therapeutic outcomes [89].

#### 4.4.8. Expanding the Role of Cosibelimab in Neoadjuvant or Adjuvant Therapy

While cosibelimab is currently being evaluated for metastatic or unresectable CSCC, its potential use in neoadjuvant (pre-surgical) or adjuvant (post-surgical) settings warrants further investigation. Evidence from other PD-1/PD-L1 inhibitors, such as pembrolizumab and cemiplimab, has demonstrated the effectiveness of these agents in both settings in cancers like melanoma and NSCLC. This provides a rationale for exploring similar applications for cosibelimab in CSCC. In the neoadjuvant setting, PD-1 inhibitors have been shown to reduce tumor size before surgery, improving the likelihood of achieving negative margins and reducing the need for extensive surgical resection. For example, pembrolizumab has been effective in the neoadjuvant treatment of melanoma, demonstrating tumor shrinkage that facilitates surgery [90]. Given the success of other PD-1 inhibitors in this context, it is plausible that cosibelimab could offer similar benefits by reducing tumor burden pre-operatively in patients with laCSCC. In the adjuvant setting, PD-1 inhibitors have been used successfully to reduce the risk of recurrence after surgery in high-risk cancers such as melanoma and NSCLC [91]. For example, cemiplimab has shown promise in reducing recurrence rates post-surgery in patients with advanced BCC, highlighting the potential for similar approaches in CSCC [67]. Cosibelimab could be explored as an adjuvant therapy in high-risk CSCC patients, particularly those with large tumors, positive margins, or perineural invasion, where the risk of recurrence is higher. Although there is currently no direct clinical evidence for cosibelimab in these specific settings, the existing data on other PD-1 inhibitors provide a strong rationale for future clinical trials. Studies focusing on cosibelimab’s use in tumor reduction before surgery and prevention of recurrence post-surgery could broaden its application beyond unresectable disease, offering an expanded therapeutic window for patients with resectable but high-risk CSCC.

## 5. Conclusions

Cosibelimab shows significant promise as a treatment for advanced CSCC, offering a novel immunotherapeutic approach for patients with limited treatment alternatives. Its dual mechanism of action, which includes PD-L1 inhibition and potential ADCC, positions cosibelimab as a unique candidate in the ICI landscape. Clinical trials have demonstrated its efficacy, with ORRs comparable to existing therapies such as pembrolizumab and cemiplimab. Additionally, its favorable safety profile, characterized by a lower incidence of severe irAEs, further supports its viability as a treatment option. Despite these advances, challenges remain, particularly with respect to resistance mechanisms and optimizing patient selection based on biomarkers. Translational research should focus on exploring cosibelimab’s potential in combination therapies, as well as its role in neoadjuvant and adjuvant settings. These approaches could improve surgical outcomes, reduce recurrence rates, and help expand the therapeutic landscape. As the field of immuno-oncology progresses, cosibelimab is poised to become an integral part of personalized treatment strategies for CSCC, ultimately improving patient outcomes.

## Figures and Tables

**Table 1:** Inclusion and exclusion criteria and subgroup analysis for the Phase I and Phase II trials of cosibelimab, excluding safety profile, trends, and efficacy outcomes [65].

Criteria/Analysis	Phase I Trial	Phase II Trial
Inclusion Criteria	- Adult patients with mCSCC (nodal and/or distant)	- Adult patients with metastatic or locally advanced CSCC unsuitable for curative surgery or radiation
	- ECOG performance status of 0 or 1	- ECOG performance status of 0 or 1
	- Adequate organ function	- Adequate organ function
	- At least one measurable lesion according to RECIST 1.1	- At least one measurable lesion according to RECIST 1.1
Exclusion Criteria	- Prior immune checkpoint inhibitor therapy	- Active or suspected autoimmune disease
	- Active or suspected autoimmune disease	- Prior exposure to anti-PD-(L)1 therapy or other immune checkpoint inhibitors
	- Immunosuppressive doses of steroids (>10 mg/day prednisone or equivalent)	- Immunosuppressive doses of steroids (>10 mg/day prednisone or equivalent)
	- Uncontrolled cardiovascular disease	- Uncontrolled cardiovascular disease
	- HIV, hepatitis B or hepatitis C virus infection	- HIV, hepatitis B or hepatitis C virus infection
	- ECOG performance status greater than 2	- ECOG performance status greater than 2
Treatment Regimen	Cosibelimab administered at 800 mg every 2 weeks	Cosibelimab administered at 800 mg every 2 weeks until progression or intolerable toxicity
Subgroup Analysis	Not explicitly mentioned in Phase I trial details	- Metastatic vs. locally advanced disease
		- ECOG performance status (34% with status 0 and 66% with status 1)
		- Age subgroups (median age of 75, 78% ≥ 65 years)
		- Sex and race (72% male, 85% White)
		- Prior systemic therapy (7% had prior systemic therapy)

mCSCC, metastatic cutaneous squamous cell carcinoma; ECOG, eastern cooperative oncology group; RECIST, response evaluation criteria in solid tumors; CSCC, cutaneous squamous cell carcinoma; HIV, human immunodeficiency virus; anti-PD-(L)1, anti-programmed cell death (ligand) 1.

**Table 2:** Comparative efficacy and safety of cosibelimab across various cancer types [4,65,69,70,71,72,73,74,75,76].

Category	CSCC-Specific Phase II Trial	Broader Phase II Trial (Various Tumor Types)	Phase I Trial (Early-Stage Study)
ORR	47.5% (CSCC patients)	47% (CSCC cohort, lower ORRs in other tumor types like NSCLC, bladder cancer)	Modest efficacy in CSCC, preliminary data
CR	7%	Not reported for CSCC	Not reported
PR	40.5%	Not specified	Early signs of PRs
PFS	12.9 months	Varies across tumor types, similar in CSCC cohort (lower PFS in NSCLC and bladder cancer)	Not reported
DOR	11.3 months	Similar to CSCC cohort	Not specified
Safety Profile	Fatigue, rash, pruritus (Grade 1/2)	Similar to CSCC cohort; irAEs such as colitis, hepatitis, pneumonitis in NSCLC and bladder cancer	Focused on safety and DLTs; no MTD
Severe Adverse Events (Grade 3 or Higher)	<10% (rare, manageable)	Similar; small subset with irAEs in NSCLC and bladder cancer	Low incidence of severe AEs, no MTD reached
irAEs	Pneumonitis, immune-mediated dermatitis	Colitis, hepatitis, pneumonitis (especially in NSCLC and bladder cancer), manageable with corticosteroids	No major immune-related toxicities observed
Management of Adverse Events	Standard immunosuppressive treatments	Pauses or corticosteroids for irAEs in NSCLC and bladder cancer	Standard interventions, no MTD reached

ORR, objective response rate; CSCC, cutaneous squamous cell carcinoma; NSCLC, non-small cell lung cancer; CR, complete response; PR, partial response; PFS, progression-free survival; DOR, duration of response; AE, adverse event; DLT, dose-limiting toxicity; MTD, maximum tolerated dose; irAE, immune-related adverse event.

**Table 3:** Comparative analysis of cosibelimab, pembrolizumab, and cemiplimab in CSCC.

Category	Cosibelimab [65]	Pembrolizumab [58,78]	Cemiplimab [58,78]
Mechanism of Action	PD-1/PD-L1 pathway (binds directly to PD-L1 on tumor cells), potential for ADCC	PD-1/PD-L1 pathway (inhibits PD-1 on T cells)	PD-1/PD-L1 pathway (inhibits PD-1 on T cells)
ORR	47.5% (Phase II)	40–50%	47%
CR Rate	7%	Lower than cemiplimab	13%
DOR	11.3 months	Similar to cemiplimab, PD-L1 dependent	16.8 months
PFS	12.9 months	Comparable to cemiplimab	18 months
Efficacy Across PD-L1 Levels	Effective across all PD-L1 levels	Better in PD-L1 high expressors (≥50%)	Effective across all PD-L1 levels
Common Adverse Events	Fatigue, rash, pruritus, diarrhea	Fatigue, rash, pruritus, diarrhea	Fatigue, rash, pruritus, diarrhea
Severe irAEs	<10% (lower incidence of Grade 3 or higher irAEs)	10–15% (similar risks of irAEs, particularly in autoimmune patients)	10–15% (higher risk of endocrinopathies)
Endocrinopathies	Lower incidence	Similar to cemiplimab	Higher incidence (requires close monitoring)
Dosing Schedule	Every 2 weeks	Every 3 or 6 weeks	Every 3 weeks
Clinical Availability	Not yet approved for widespread use	Widely available and in clinical practice	Widely available and in clinical practice
Long-Term Survival Data	Not fully available, but early data shows promise	Median OS 22 months, median PFS 18.4 months	Median OS 19 months, median PFS 16.8 months
Efficacy in Long-Term Control	Potential for long-term responses, needs validation	Durable responses in PD-L1 high expressors	Consistent long-term control, effective across PD-L1 levels

PD-1, programmed cell death protein 1; PD-L1, programmed cell death ligand 1; ADCC, antibody-dependent cellular toxicity; CR, complete response; DOR, duration of response; PFS, progression-free survival; irAE, immune-related adverse event; OS, overall survival.

## Data Availability

No new data were created in this study.

## Abbreviations

The following abbreviations are used in this manuscript:

ADCC	Antibody-Dependent Cellular Cytotoxicity
AK	Actinic Keratosis
AKT	Protein Kinase B
BCC	Basal Cell Carcinoma
BLA	Biologics License Application
CR	Complete Response
CSCC	Cutaneous Squamous Cell Carcinoma
DLT	Dose-Limiting Toxicity
DOR	Duration of Response
EMA	European Medicines Agency
ERK	Extracellular Signal-Regulated Kinase
FDA	U.S. Food and Drug Administration
ICB	Immune Checkpoint Blockade
irAE	Immune-Related Adverse Event
laCSCC	Locally Advanced Cutaneous Squamous Cell Carcinoma
MAPK	Mitogen-Activated Protein Kinase
mCSCC	Metastatic Cutaneous Squamous Cell Carcinoma
MDSC	Myeloid-Derived Suppressor Cell
MTD	Maximum Tolerated Dose
NK	Natural Killer (cells)
NSCLC	Non-Small Cell Lung Cancer
ORR	Objective Response Rate
OS	Overall Survival
PD-1	Programmed Cell Death Protein-1
PD-L1	Programmed Death-Ligand 1
PFS	Progression-Free Survival
PI3K	Phosphoinositide 3-Kinase
PKB	Protein Kinase B
PR	Partial Response
RECIST	Response Evaluation Criteria in Solid Tumors
ROS	Reactive Oxygen Species
SCC	Squamous Cell Carcinoma
Treg	Regulatory T Cell
